# Suggestion of self-(in)coherence modulates cognitive dissonance

**DOI:** 10.1371/journal.pone.0202204

**Published:** 2018-08-30

**Authors:** Joshua Hagège, Mariam Chammat, Caroline Tandetnik, Lionel Naccache

**Affiliations:** 1 dINSERM, U 1127, Paris, France; 2 Institut du Cerveau et de la Moelle épinière, ICM, PICNIC Lab, Paris, France; 3 Sorbonne Universités, UPMC Univ Paris 06, Faculté de Médecine Pitié-Salpêtrière, Paris, France; 4 AP-HP, Groupe hospitalier Pitié-Salpêtrière, Department of Neurology, Paris, France; 5 AP-HP, Groupe hospitalier Pitié-Salpêtrière, Department of Neurophysiology, Paris, France; Technion Israel Institute of Technology, ISRAEL

## Abstract

While cognitive dissonance is an influential concept of social psychology, its relations with consciousness and episodic memory remain strongly debated. We recently used the free-choice paradigm (FCP) to demonstrate the crucial role of conscious memory of previous choices on choice-induced preference change (CIPC). After choosing between two similarly rated items, subjects reevaluated chosen items as more attractive, and rejected items as less attractive. However such a CIPC was present exclusively for items that were correctly remembered as chosen or rejected during the choice stage, both in healthy controls and in amnesic patients. In the present work, we show that CIPC can be modulated by suggestive quotes promoting self-coherence or self-incoherence. In addition to the crucial role of memory of previous choices, we discovered that memory of the suggestive quotes was correlated to the modulation of CIPC. Taken together these results suggest that CIPC reflects a dynamic homeostatic regulation of self-coherence.

## Introduction

While it is very intuitive that our current preferences and values influence our future choices, the reverse causality is much less easy to predict and explain. However several empirical observations have confirmed that our previous choices can indeed influence our values and preferences. Such a reverse causality has been coined “cognitive dissonance” resolution, and is one of the most influential concept of social psychology since the seminal work of Festinger in the 50s [1]. For instance, in the free-choice paradigm (FCP), after choosing between two similarly rated items, subjects reevaluate chosen items as more attractive and rejected items as less attractive [2]. This effect is measured as a spreading of alternatives (difference of ratings 1 and 2 according to chosen/rejected status of the item). One key scientific question deals with the relation between choice-induced preference change (CIPC) on the one hand, and episodic memory of previous choices on the other hand: is this phenomenon dependent or independent from memory of past choices? (see [3, 4] for a recent review on this issue). This debated question became complicated because of subtle statistical artefacts that were discovered and confirmed only recently [5–7]: spreading of alternatives could be observed in the absence of genuine preference change. A univocal way to control for this artifact consists in using a new control condition during which subjects perform their two ratings before the choice stage (RRC), in addition to the traditional rating/choice/rating sequence (RCR).

Taking these artefacts into account, we recently solved this debate about CIPC and memory, by reproducibly showing that CIPC occurs exclusively for items that were correctly remembered as chosen or rejected during the choice stage [3, 4]. Moreover, we used a combination of fMRI and intracranial electrophysiological recordings to reveal a modulation of left hippocampus activity, a hub of episodic memory retrieval, immediately before the occurrence of CIPC during item reevaluation (Rating 2 stage). We also showed that CIPC is absent in amnesic patients for forgotten items.

Inspired by this demonstration that CIPC depends on conscious episodic memory, and therefore that it is not an automatic and irrepressible process, we decided to address the following question: could CIPC be modulated by suggestive quotes promoting self-coherence of self-incoherence?

To our knowledge, very few studies explored this issue in other paradigms traditionally associated with cognitive dissonance, such as the classical “counter-attitudinal statement” paradigm, in which subjects asked to write a counter-attitudinal essay tend to change their mind accordingly [8]. Bator and Cialdini [9] found that only participants whose consistency motives had been activated by the exposition to an alleged letter valuating consistency, showed greater attitude following a counter-attitudinal statement. Similarly, Cialdini et al. [10] showed that when a specific norm was made salient to the participants, their behavior tended to follow that norm. Note however that these two studies did not explore the FCP in which the issue of automaticity is still strongly debated. This scientific question is also theoretically important, because FCP constitutes the subdomain of cognitive dissonance for which neural mechanisms at work have been investigated the most [6, 11–16].

If CIPC could be modulated by suggestion, this would further demonstrate its non-automatic nature. Such a result obtained by manipulating self-coherence would also further confirm the importance of frontal lobe mediated executive control mechanisms at work in CIPC [6, 11, 12, 14–16].

In the present study we provide such a demonstration, and reveal that episodic memory of the suggestive quotes is correlated to the modulation of CIPC in the FCP.

## Materials & methods

### Ethics statement

This experiment has been approved by the Pitié-Salpêtrière ethical committee. All the 73 subjects gave their written informed consents, and were paid 10 Euros to participate in the experiment. All investigations were conducted according to the principles expressed in the Declaration of Helsinki.

### Participants

27 healthy participants were included in the Control group (12 women; age M = 23.25 years old; STD = 3.02), 24 healthy participants were included in the Coherence group (13 women; age M = 22.95; STD = 2.83), and 22 healthy participants were included in the Incoherence group (11 women, age M = 23.36; STD = 3.07) leading to a total of 73 participants. They reported normal, or corrected-to-normal, visual acuity. This sample size was estimated according to our two previous studies using the very same free-choice paradigm ^3,4^, and to other studies from other groups.

### Stimuli

Stimuli consisted of 160 (80 for each part: A & B) equalized images of holiday destination (subtended 5.3° of the visual field), with the name of the destination written underneath it, on the center of the screen (font size = 30). In 'Rating' blocks, one image appeared at the center of the screen in each trial, whereas in 'Choice' blocks, two targets were presented 4.8° off-center, to the left and to the right, in each trial.

**Fig 1:**
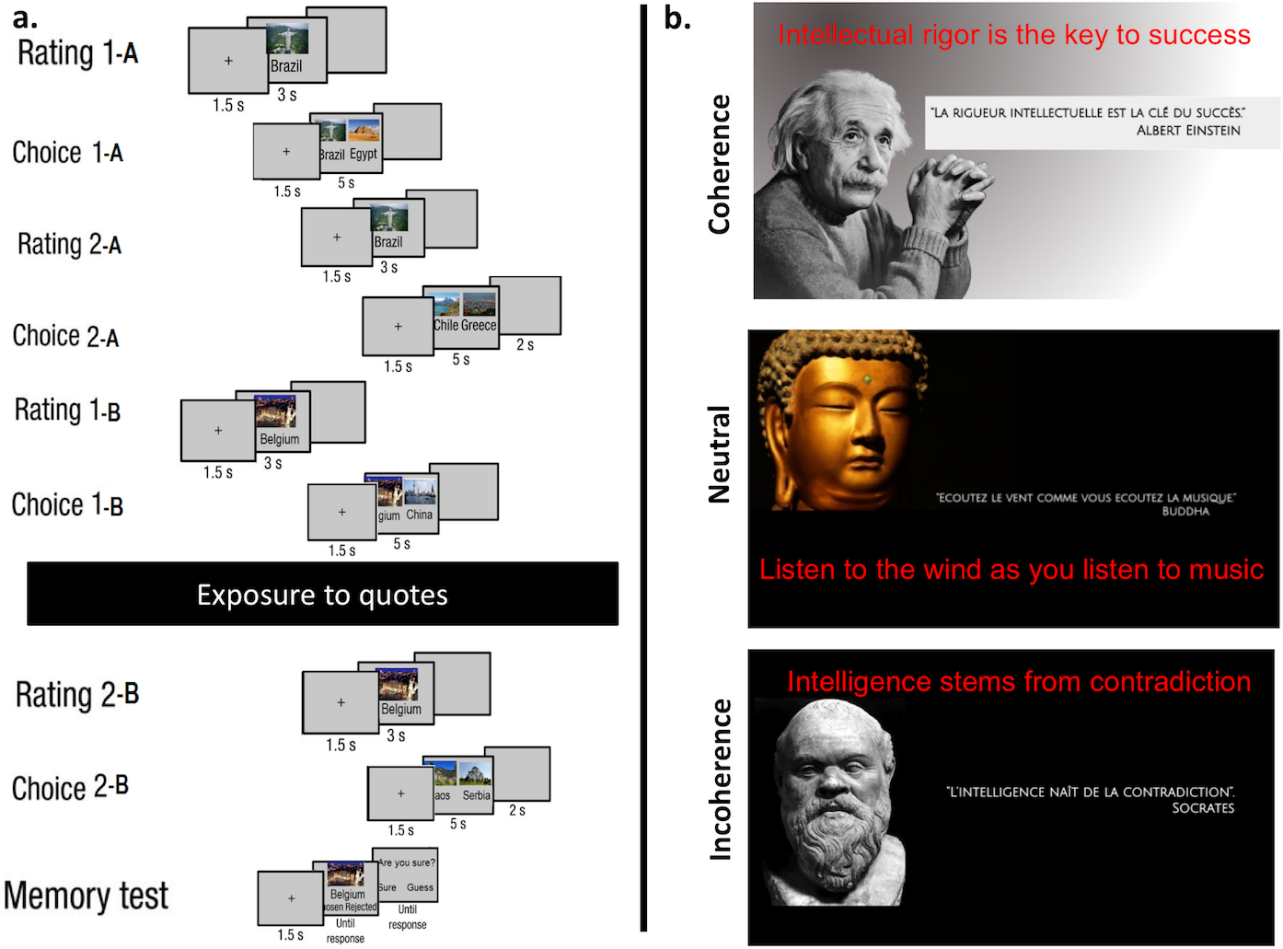
Experimental paradigm. (a) The experiment was composed of 9 blocks separated by a quote exposure period occurring after the 6^th^ block (Choice 1-B): 4 blocks of rating (Rating 1-A, Rating 2-A, Rating 1-B, Rating 2-B), 4 blocks of choice (Choice 1-A, Choice 2-A, Choice 1-B, Choice 2-B), and a memory block for every groups of subject (Control—Coherence—Incoherence). Items were divided in two subsets for Part A and Part B respectively. Part A was used as a control test of CIPC, and included both the RCR condition of interest, and the RRC control condition. Subjects were then engaged in Part B that was the genuine test of interest in which we predicted to modulate CIPC by quote exposure. Part B started with a rating block (Rating 1-B), followed by the Choice 1-B block. A confederate then entered in the experimental room requesting the examiner to come with him to solve an urgent problem. The examiner accepted to accompany the confederate but asked her to pause the ongoing experiment. Actually, the confederate set up a desktop background with one of the 3 quote categories (control-coherence-incoherence) that would then be presented on the screen for 3 minutes. This design allowed a clear double blind experiment. Images were free of use for commercial usage. (b) Three examples of desktop backgrounds used including the quotes with their English translation. All images displayed in this figure are free of use for commercial usage (Rio de Janeiro Corcovado mountain by Artyominc https://fr.wikipedia.org/wiki/Fichier:Christ_on_Corcovado_mountain.JPG (CC BY-SA 3.0 https://creativecommons.org/licenses/by-sa/3.0/deed.en); Gizah pyramids by Ricardo Liberato https://en.wikipedia.org/wiki/File:All_Gizah_Pyramids.jpg (CC BY-SA 2.0 https://creativecommons.org/licenses/by-sa/2.0/deed.en); Santiago Chile by Patrick Coe https://commons.wikimedia.org/wiki/File:Santiago_Chile.jpg (CC BY 2.0 https://creativecommons.org/licenses/by/2.0/deed.en); Greek landscape https://pixabay.com/fr/gr%C3%A8ce-mer-vue-sur-la-mer-sud-905559/ is in the public domain with no attribution required (CC0 https://creativecommons.org/publicdomain/zero/1.0/deed.en); Belgium urban landscape https://pxhere.com/fr/photo/419689 (CC BY 2.0 https://creativecommons.org/licenses/by/2.0/) China Urban landscape https://pixabay.com/fr/shanghai-bund-la-chine-ville-1484452/ is in the public domain (CC0 https://creativecommons.org/publicdomain/zero/1.0/deed.en); Vicinities NongFa lake in Laos by Aleksey Gnilenkov https://commons.wikimedia.org/wiki/File:Vicinities_NongFa_lake_in_Laos_(5514443712).jpg (CC BY 2.0 https://creativecommons.org/licenses/by/2.0/deed.en); Temple of Saint Sava by Borisa Zivkovic is in the public domain (CC0 https://creativecommons.org/publicdomain/zero/1.0/deed.en); Gouden Buddha by Mwibawa https://commons.wikimedia.org/wiki/File:WLANL_-_mwibawa_-_Gouden_Buddha_(1).jpg (CC BY-SA 2.0 https://creativecommons.org/licenses/by-sa/2.0/deed.en); Albert Einstein by Emilio Segrès https://www.flickr.com/photos/sfjalar/2931059489 CC BY 2.0 https://creativecommons.org/licenses/by/2.0/); Lysippos, Socrates Statue at the Louvre https://commons.wikimedia.org/wiki/File:Socrates_statue_at_the_Louvre,_8_April_2013.jpg (CC BY 2.0 https://creativecommons.org/licenses/by/2.0/deed.en).

We used fictional quotes quotes that we attributed to Socrates, Buddha and Einstein in order to manipulate subjects’ attitude toward self-coherence according to 3 conditions (3 quotes for each condition, see Fig 1 and 1 Quotes for a list of the 9 quotes with their alleged author). In the self-coherence promoting condition, one of the quotes was: "Intellectual rigor is the key to success" attributed to Albert Einstein. In the self-incoherence promoting condition, one of the quotes was: "Intelligence stems from contradiction" attributed to Socrates. In the neutral control condition, quotes emphasize poetry and music, and one of the three quotes was: "Listen to the wind as you listen to music" attributed to Buddha. Each quote was presented twice pseudo-randomly (no immediate repetition of the same quote) on the desktop background immediately after Choice-1B block, for a duration of 30 seconds, leading to a total quote exposure time of 3 minutes. Each quote was displayed in association with a picture of its alleged author.

### Procedure

The experiment was composed of 9 blocks separated by a quote exposure period occurring after the 6^th^ block (Choice 1-B; see Fig 1): 4 blocks of rating (Rating 1-A, Rating 2-A, Rating 1-B, Rating 2-B), 4 blocks of choice (Choice 1-A, Choice 2-A, Choice 1-B, Choice 2-B), and a memory block for every group of subject (Control—Coherence—Incoherence). Items were divided in two subsets for Part A and Part B respectively. Part A was used as a control test of CIPC, and included both the RCR and RRC conditions, while Part B corresponded to the quote exposure manipulation phase.

The experiment started with a first rating (Rating 1-A), which included 80 trials. Each trial began with a fixation point presented during 1.5 second. Then one vacation destination was centrally presented for 3 seconds, followed by a blank screen lasting until subjects responded manually. They were requested to report how much they would like to spend their vacation in this destination using an eight-point scale (1 = ‘I do not want to go there at all’, 8 = ‘I definitely want to go there’). Subjects responded using the 1–8 number pad buttons of a regular keyboard. This first block was followed by a choice (Choice 1-A for the RCR condition), which included 20 trials (40 destinations were coupled). Participants were presented with pairs of destinations they had rated equally (difference of R1 scores≤ 1) and had to indicate with a button press at which one they would rather take their vacation. Note that all individual medians of R1 scores differences were null.

In this choice block, every trial started with a fixation point presented during 1.5 second. Then, two destinations were presented side-by-side for 5 seconds. Subjects had to report manually their choice using the left/right arrows keyboard keys. Importantly, destinations were coupled according to Rating 1-A block. Each couple of destinations was composed of destinations rated similarly. Then, subjects had to perform another block of rating (Rating 2-A) on the same images as in the Rating 1-A phase. This Rating 2-A was followed by a new choice (Choice 2-A for the RRC condition). This time, subjects had to perform a choice between the other 40 destinations that had not showed during Choice 1-A. In the same way, those destinations were coupled according to Rating 1-A. This block was the last one of the part A.

Part B of the experiment started with a rating block (Rating 1-B). This block followed the very same description that Rating 1-A block except that 80 new destinations were concerned. Following Rating 1-B stage, subjects were engaged in the Choice 1-B block which was structured similarly to the Choice 1-A block except that destinations were coupled according to Rating 1-B block. After this Choice 1-B, the manipulation period started. A confederate then entered in the experimental room requesting the examiner to come with him to solve an urgent problem. The examiner accepted to accompany the confederate but asked her to pause the ongoing experiment. Unbeknownst to the subject, the confederate set up a desktop background with one of the 3 quote categories (Coherence/Incoherence/Control) that would then be presented on the screen for 3 minutes (3 quotes per category were presented twice for 30 seconds on each presentation; see SI1 for the list of quotes used), and both the examiner and the confederate left the room. This design allowed a clear double blind experiment whereby neither subjects nor experimenter knew which category the subjects were in. Upon the return of the examiner, subjects had to perform another block of rating (Rating 2-B). They had to rate the same destinations that has been rated during Rating 1-B. This Rating 2-B was followed by a new choice (Choice 2-B). This time, subjects had to perform a choice between the other 40 destinations that had not been shown during Choice 1-B. In the same way, those destinations were coupled according to Rating 1-B.

The last block of the experiment was a memory block. Participants were asked, for each destination whether they had chosen or rejected it. Then, they were asked to report their confidence in their response. For both questions, subjects had to answer manually using the left/right keyboard arrows. As we were interested in testing whether subjects remembered the episode of the choice they made, we considered items as remembered only if subjects correctly reported whether they had chosen or rejected each of the two coupled items.

At the end of the experiment subjects filled out a debriefing form in which they were asked to recall and write down the three quotes they were exposed to, and to report whether they interpreted the experiment interruption as intentionally planned by the experimenters. We then calculated a quote memory score for each subject using the following criteria: 1 point if the quote was remembered; 0.5 point if some crucial words were remembered; 0.25 point if only the name of the alleged author of the quote was remembered; 0 point if nothing relevant was remembered. Note that spreading of alternatives is computed as (second rating for a chosen item–first rating for a chosen item)–(second rating for a rejected item–first rating for a rejected item).

### Statistics

We used linear mixed-effects models that offer the possibility to handle the heteroscedasticity related to the unbalanced number of items in each condition [17]. Significance of the fixed effects was assessed using the Kenward-Roger approximation for degrees of freedom of the denominator, with the ‘lmerTest’ package in R. The experimental dataset is available in S1 Experimental Dataset.

## Results

**Fig 2:**
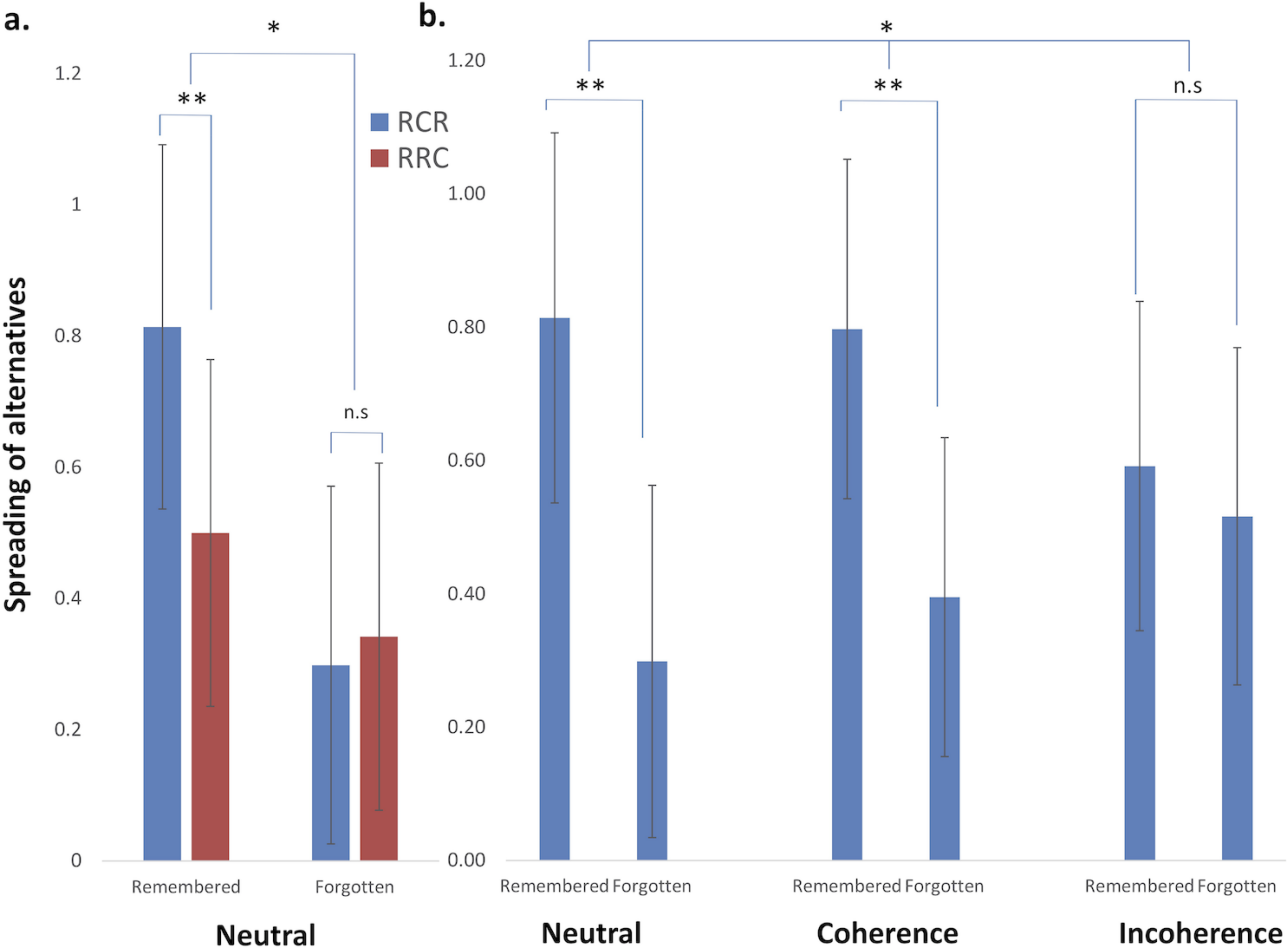
Exposure to quotes promoting self-incoherence decreased CIPC. Spreading of alternatives is computed as (second rating for a chosen item–first rating for a chosen item)–(second rating for a rejected item–first rating for a rejected item). (a) In the Neutral condition, we replicated the episodic memory dependent CIPC effect, as a significant interaction between choice-memory (Part B) and RCR/RRC condition, and confirmed that a RCR>RRC effect was present exclusively for remembered items (** for 0.001≤p<0.01; * for 0.01≤p<0.05). In all figures, error bars correspond to standard errors of the mean. (b) Comparison of spreads in the RCR condition differed across the 3 conditions: while exposure to neutral and coherence quotes showed similar CIPC effects, exposure to incoherent quotes abolished the CIPC effect.

We first replicated our previous finding about the impact of memory on choice induced preference change in the ‘Neutral’ group [3, 4], by observing a significant interaction between condition (RCR/RRC) and choice memory (remembered vs forgotten choices; F(1,1073.8) = 5.44; p = 0.02; see Fig 2). A choice induced preference change (RCR>RRC) was present exclusively for remembered choice trials (see Fig 2A).

We then confirmed the predicted impact of quotes on choice induced preference change, as a significant interaction between group (‘Coherence’, ‘Neutral’, ‘Incoherence’), and choice memory (remembered vs forgotten choices) on RCR trials (F(2,1436) = 3.88, p = 0.02; see Fig 2B). Note that the triple interaction between group, choice memory and condition (RCR/RRC) is not valid here given that choices were made before exposure to quotes in the RCR condition, and after it in the RRC condition (see below). Post-hoc contrasts revealed that this modulation of preference change by quotes was driven by the ‘Incoherence’ group in which no difference was observed between remembered and forgotten trials (F(1,438) = 0.38; p = 0.54), whereas this difference was present in the two other groups (‘Neutral’ group: F(1,526) = 21.8; p <10^−5^ and for the ‘Coherence’ group: F(1,476) = 15.6; p <10^−4^). Importantly, the impact of quotes on CIPC was not affected by participants’ interpretation of experiment interruption: we performed an ANOVA on RCR spread including this new factor (i.e. categorizing participants as considering the interruption of the experiment as planned (N = 11) or unplanned (N = 62)). The triple interaction between this factor and Group(Coherence/Incoherence/Neutral) and Choice memory(2) factors was not significant (F(2,1429) = 1.86; p = 0.16).

**Fig 3:**
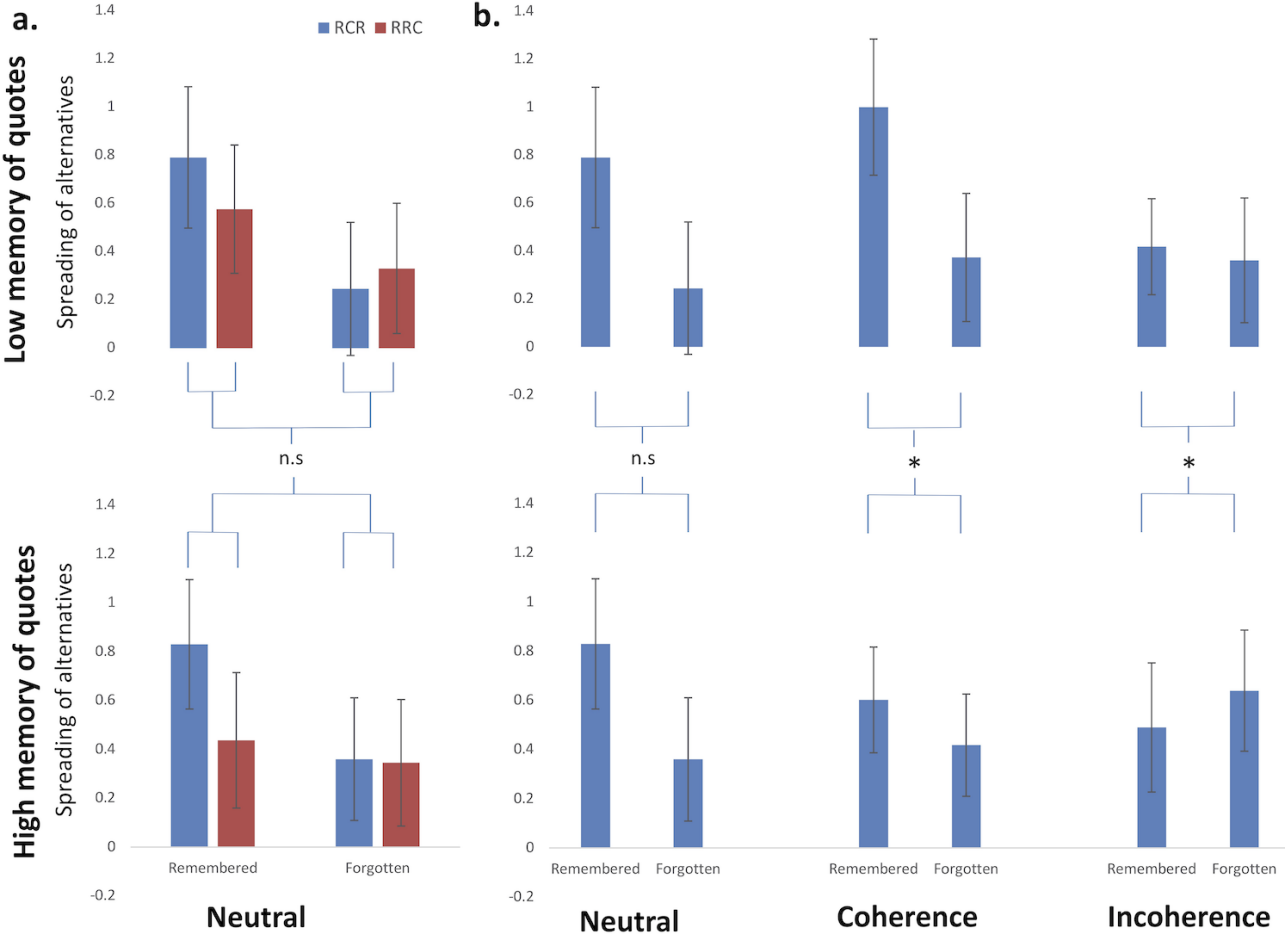
Quote-mediated modulation of CIPC is correlated to memory of quotes. (a) In the neutral quotes condition, the usual episodic dependent CIPC effect was not modulated by memory of quotes. (b) In contrast, comparisons of RCR spreads revealed a modulation of CIPC in relation to quotes memory in the two conditions of interest (* for 0.01≤p<0.05). In the incoherence promoting quotes condition, the spread was significantly lower for subjects who remembered well the quotes than for those that did not. In the coherence promoting quotes condition, the spread was significantly larger for subjects who did not remember well the quotes, suggesting a coherence exerted more on Rating 1 responses than on Choice stage decisions.

We then probed the potential impact of episodic memory of quotes on quote-induced modulation of preference change. We split each experimental group in two subgroups according to subjects’ individual score on the quotes memory task, relative to the median value of this score calculated across the whole group. As for the previous analysis we could first control data quality by computing the 3-factors ANOVA on the ‘Neutral’ group only. As expected, the behavioral signature of memory dependent choice-induced preference change was not affected by the memory for neutral quotes (F(1,1070) = 0.037; p = 0.84; see Fig 3A). We then analyzed the spreads within each experimental group with an ANOVA crossing choice memory and quotes memory (see Fig 3B) on RCR trials only. Again, the larger spread observed for remembered choices as compared to forgotten choices was not affected by quotes memory in the ‘Neutral’ group (F(1.528) = 0.1; p = 0.75). Crucially, an inversion of this effect was found in the ‘Incoherence’ group: while the spread was larger for remembered than for forgotten trials for the low quotes memory subgroup, the polarity of this difference was inverted for the high memory subgroup (F(1,436) = 4.1; p = 0.04; see Fig 3 right panel). The same analysis conducted in the ‘Coherence’ group led to an unexpected result. An interaction was observed with a larger effect for the low quotes memory subgroup as compared to high quotes memory subgroup (F(1,474) = 5.15; p = 0.02).

This result could be explained by a process of coherence exerted on rating 1 scores rather than on choice performance only. Post-hoc tests strengthened this interpretation by revealing that memory of citations impacted half-spread (R2-R1 for a given item) of chosen items in the predicted direction: the more subjects remembered the quotes, the closer were R2 and R1 (mean half-spread = 0.13 for high memory, versus 0.43 for low-memory; p = 0.03). It is noticeable that this impact was not observed on rejected items (p = 0.34). This asymmetry was confirmed on a dedicated ANOVA calculated on half-spreads, as a significant interaction between chosen/rejected and low versus high memory of citations (F(1,934) = 7.86; p = 0.005). Note that this strategy,—observed in the ‘Coherence’ group -, of privileging coherence on rating 1 score rather than on choice stage performance, would not lead to similar result if applied in the ‘Incoherence’ group.

In order to better understand the impact of quotes, we then analyzed the RRC trials. As expected, no significant interaction between group and choice memory was observed (F(2,1422) = 0.88, p = 0.41), and we then focused on the impact of quotes. A significant interaction was found between group and quotes memory factors (F(2,67) = 3.41, p = 0.04). Post-hoc analyses revealed that while quotes memory did not modulate RRC spreads in the neutral (F(1,25) = 0.46,p = 0.50) and incoherence (F(1,20) = 0.87, p = 0.36) groups, a significant effect was present for the coherence group exclusively (F(1,22) = 8.42,p = 0.008). A smaller spread was observed for well memorized quotes. This result strengthens our proposed interpretation for RCR trials: when participants memorize the quotes, they tend to exert their coherence on the R2/R1 matching rather than on R2/choice matching.

Our experimental design also aimed at collecting an additional control condition for each subject by using the ‘Part A’ data (see Fig 1 and M&M). However, subjects forgot the choices made during ‘Part A’ much more than the choices made during the ‘Part B’ suggestion phase (‘Part B’; (t(38) = -5.2, p<10^−5^). This forgetting effect was most probably due to the long time interval (>30 minutes) separating RCR choices of ‘Part A’ from the memory task, and by the existence of memory interference related to the 4 distinct choice phases (RRC and RCR in both parts).). In spite of this forgetting effect, we confirmed the absence of difference of CIPC across the three groups in Part A: there was no significant triple interaction between Group, Condition and Memory factors on spread values (F(2,2900) = 0.58; p = 0.56).

Finally, too few choices were confidently remembered in the present study, prevailing any analysis of confidence. This was probably due to the high-load of items, choices, experimental stages and experiment duration, as compared to our previous reports in which both objective responses and subjectively confident measures of episodic memory were very close and led to similar results [3].

Taken together, our results demonstrate that CIPC can be manipulated by suggestive instructions, and that this manipulation is dependent of explicit memory.

## Discussion

We discovered a significant impact of quotes promoting incoherence as a general cognitive posture, on CIPC, as compared to neutral quotes and to quotes promoting coherence. This suggestibility effect was even stronger for those individuals who consciously remembered the quotes the best. In agreement with our previous finding on the necessary role of episodic memory of previous choices on CIPC [3, 4], our current study enriches the range of cognitive factors that can affect CIPC, and further confirms that CIPC is not an automatic process. Interestingly, episodic memory seems to convey at least two information that can affect CIPC: memory of previous choices, and memory of current external suggestion. Future neurophysiological works could detail how these distinct types of information are encoded in the episodic memory network, and how they are used by executive control, decision-making and value-system networks.

The absence of difference between neutral quotes and coherence promoting quotes may be explained by two non-mutually exclusive hypotheses. First, this absence of difference may reflect a floor effect due to a general bias toward search of coherence: if individuals are spontaneously looking for coherence,—as reflected by the spontaneous CIPC effect -, we may have missed an increase of this trend. Additional experiments using more efficient quotes or longer exposure to quotes may reveal a possible increase of CIPC. An alternative hypothesis of this absence of difference between neutral and coherence promoting quotes may be related to the impact of quotes: as suggested by our additional analyses, quotes may have emphasized both a coherence effect related to previous choices (and leading to an increase CIPC), but also a coherence effect related to Rating 1 (and leading to a null CIPC). Therefore, we may still increase CIPC with coherence-promoting quotes, provided that they are more directive and more clearly addressed to previous choices.

We conclude by raising the puzzling issue of consciousness and agentivity during CIPC: if CIPC only occurs for consciously remembered items, and if CIPC is more subject to suggestion when we remember better the instructions, one may wonder up to which point CIPC is a voluntarily/involuntarily and conscious/unconscious process. Is CIPC a non-reportable non-conscious process that requires an information (previous choice or current instruction) to be consciously accessed first? Or is CIPC accessible to conscious introspection, and even subjectively considered as an intentional act within the experimental context? Future psychological studies should better explore these two questions, now that the impact of episodic memory and of suggestion have been reliably shown on CIPC.

## Supporting information

S1 Experimental Dataset (ZIP)

S1 Quotes (DOCX)
