# Supplementary material for: Suggestion of self-(in)coherence modulates cognitive dissonance
Source: PLoS One. 2018 Aug 30;13(8):e0202204. doi: 10.1371/journal.pone.0202204 (PMC6116930; doi:10.1371/journal.pone.0202204)
Supplement: S1 Quotes — (DOCX) [file pone.0202204.s002.docx]

**S1 Quotes.**

For the Coherence group :

"La rigueur intellectuelle est la clé du succès." Einstein

"Intellectual rigor is the key to success." Einstein

"La dignité n'existe que dans le respect de ses propres actions." Socrates

"Dignity only exists in the respect of one's own actions." Socrates

"Le passé ne peut être changé, oublié, édité ou effacé. Tu te dois de l'accepter." Buddha

"Past cannot be changed, forgotten, edited or erased. You must accept it." Buddha

For the Incoherence group:

"L'imbécile n'est pas celui qui a tort, mais celui qui refuse de changer d'avis." Einstein

"The fool is not the wrong, but the one who refuse to change his mind." Einstein

"L'intelligence naît de la contradiction." Socrates

"Intelligence stems from contradiction" Socrates

"Sois spontané et tu seras toi-même." Buddha

"Be spontaneous and you will be yourself." Buddha

For the Control group:

"La musique est une mathématique sonore." Einstein

"Music is sonorous mathematics." Einstein

"Un poème prend sens dans la voix du poète." Socrates

"A poem makes sense in the poet's voice." Socrates

"Ecoutez le vent comme vous écoutez la musique." Buddha

"Listen to the wind as you listen to music." Buddha
